# Molecular docking data of piperine with Bax, Caspase 3, Cox 2 and Caspase 9

**DOI:** 10.6026/97320630016458

**Published:** 2020-06-30

**Authors:** Chandrasekaran Kirubhanand, Jayaraman Selvaraj, Umapathy Vidhya Rekha, Veeraraghavan Vishnupriya, Devarajan Nalini, Surapaneni Krishna Mohan, Periyasamy Vijayalakshmi, Manikkam Rajalakshmi, Rajagopal Ponnulakshmi

**Affiliations:** 1 Department of Anatomy, All India Institute of Medical Sciences, Nagpur, India; 2 Department of Biochemistry, Saveetha Dental College and Hospitals, Saveetha Institute of Medical and Technical Sciences, Saveetha University, Chennai - 600 077, India; 3 Department of Public Health Dentistry, Sree Balaji Dental College and Hospital, Pallikaranai, Chennai-600 100, India; 4 Central Research Laboratory, Meenakshmi Ammal Dental College, Meenakshi Academy of Higher Education and Research (Deemed to be University), Chennai-600 095, India; 5 Panimalar Medical College Hospital and Research Institute, Varadharajapuram, Poonamallee, Chennai-600 123, Tamil Nadu, India; 6 DBT-BIF Centre, PG and Research Department of Biotechnology and Bioinformatics, Holy Cross College (Autonomous), Trichy, Tamilnadu, India; 7 Central Research Laboratory, Meenakshi Academy of Higher Education and Research (Deemed to be University), West K. K. Nagar, Chennai-600 078, India

**Keywords:** Piperine, molecular docking, Bax, Caspase 3, Cox 2, Caspase 9

## Abstract

Several apoptotic signalling proteins such as Bax, Caspase 3, Cox 2 and Caspase 9 are known to be associated with colorectal cancer (CRC). It is of interest to study the interaction of these proteins with piperine a known drug candidate. We document the binding energy, hydrogen bond interaction and hydrophobic interaction between the piperine and apoptotic proteins for further consideration.

## Background

Apoptosis (programmed cell death) and its associated signalling proteins are linked to drug discovery in cancer [1-3]. Several apoptotic signalling proteins such as Bax [4], Caspase 3 [5], Cox 2 [6] and Caspase 9 [7] are known to be associated with colorectal cancer (CRC).It is of interest to study the interaction of these proteins with piperine a known drug candidate [8].

## Materials and Methods:

### Preparation of receptors structures for docking:

The data for 3D structures of proteins [9] for BAX (PDB ID: 4S0O), caspase-3 (PDB ID: 5I9B), cox-2 (PDB ID: 1CX2), caspase-9 (PDB ID: 2AR9) were used in this study.

### Ligand preparation:

Data for piperine was downloded from the pubchem database in SDF format and it was transformed as PDB file format using the Online Smile Translator. Energy minimizations of ligands were completed using the ChemBio 3D Ultra 12.0 using standard protocols.

### Molecular docking:

Molecular docking of piperine with Bax, Caspase 3, Cox 2 and Caspase 9 was completed using PatchDock [10,11] using standard procedures. The interaction between piperine and Bax, Caspase 3, Cox 2 and Caspase 9 was analyzed using Ligplot (https://www.ebi.ac.uk/thornton-srv/software/LIGPLOT/).

## Results and discussion:

**Figure 1:**
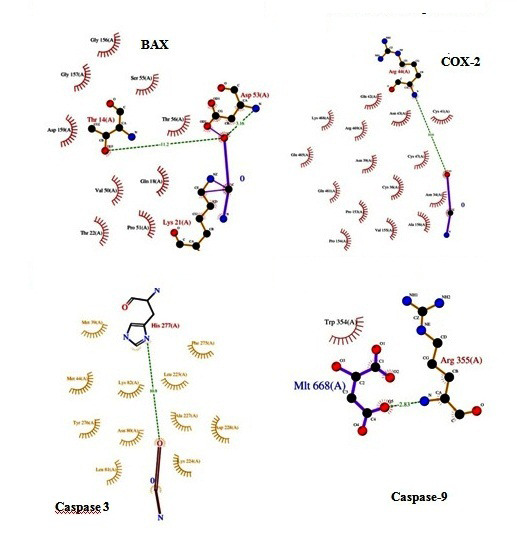
Molecular docking analysis of Bax, Caspase 3, Cox 2 and Caspase 9 with piperine is shown.

BAX, Cox-2 and Caspase 3 and 9 docked with piperine having scores 3824, 5042, 4174 and 4988 kcal/mol respectively (Table 1). The table also provides data for interacting residues with respective atomic contacts. The interaction of Bax [12,13], Cox 2 [14], Caspase 3, and Caspase 9 [15] with piperine is illustrated in (Figure 1). The interactions shows optimal binding features [16-17] for further consideration of piperine as a potential drug candidate in colon cancer research and development.

## Conclusions:

The molecular docking analysis data of selected colon cancer linked proteins such as BAX, COX-2 Cas 3 and Cas 9 with piperine is reported for further consideration.

## Figures and Tables

**Table 1:** The molecular docking analysis data of BAX, COX-2 Cas 3 and Cas 9 with piperine

S. No	Protein name	Score Kcal/mol	ACE	Residues	Ligand Atom	H-bond distance	No of non-bonded interaction
1	Bax	3824	-108.98	THR 14	OG-O	3.3	72
				ASP 53	N-O	3.16	
2	Caspase 3	4174	-187.4	HIS 277	ND-O	2.7	48
3	Cox 2	5042	-219.53	ARG 44	N-O	2.49	63
4	Caspase 9	4988	-114.31	ARG 355	N-O	2.83	10
